# Power generation in microbial fuel cells using platinum group metal-free cathode catalyst: Effect of the catalyst loading on performance and costs

**DOI:** 10.1016/j.jpowsour.2017.12.017

**Published:** 2018-02-28

**Authors:** Carlo Santoro, Mounika Kodali, Sergio Herrera, Alexey Serov, Ioannis Ieropoulos, Plamen Atanassov

**Affiliations:** a Department of Chemical and Biological Engineering, Center Micro-Engineered Materials (CMEM), MSC01 1120 University of New Mexico Albuquerque, New Mexico 87131, USA; b Bristol BioEnergy Centre, Bristol Robotics Laboratory, T-Block, UWE, Coldharbour Lane, Bristol BS16 1QY, UK; c Biological, Biomedical and Analytical Sciences, UWE, Coldharbour Lane, Bristol BS16 1QY, UK

**Keywords:** ORR catalysts, PGM-free, Rotating ring disk electrode, Microbial fuel cell, Power generation, Cost assessment

## Abstract

Platinum group metal-free (PGM-free) catalyst with different loadings was investigated in air breathing electrodes microbial fuel cells (MFCs). Firstly, the electrocatalytic activity towards oxygen reduction reaction (ORR) of the catalyst was investigated by rotating ring disk electrode (RRDE) setup with different catalyst loadings. The results showed that higher loading led to an increased in the half wave potential and the limiting current and to a further decrease in the peroxide production. The electrons transferred also slightly increased with the catalyst loading up to the value of ≈3.75. This variation probably indicates that the catalyst investigated follow a 2x2e^−^ transfer mechanism. The catalyst was integrated within activated carbon pellet-like air-breathing cathode in eight different loadings varying between 0.1 mgcm^−2^ and 10 mgcm^−2^. Performance were enhanced gradually with the increase in catalyst content. Power densities varied between 90 ± 9 μWcm^−2^ and 262 ± 4 μWcm^−2^ with catalyst loading of 0.1 mgcm^−2^ and 10 mgcm^−2^ respectively. Cost assessments related to the catalyst performance are presented. An increase in catalyst utilization led to an increase in power generated with a substantial increase in the whole costs. Also a decrease in performance due to cathode/catalyst deterioration over time led to a further increase in the costs.

## Highlights

• PGM-free different loadings were investigated in RRDE and MFC.
• In RRDE, increase in loading led to better performance and lower H_2_O_2_ produced.
• A 2x2e^−^ transfer mechanism can be speculated.
• Power densities in MFCs varied between 90 ± 9 μWcm^−2^ and 262 ± 4 μWcm^−2^.
• Increase in power density coincides with increase in the loading and in the cost.

## 1 Introduction

Cathode reduction is the bottleneck reaction in several of the electrochemical and bio-electrochemical systems [1], [2], [3], [4]. Despite several oxidants are proposed and used [5], the majority of those systems utilize oxygen as final electron acceptor due to unique characteristics that it possesses such as high potential towards the reduction reaction, natural high availability, low cost and the fact that is continuously naturally provided to the cathode without the necessity of supplying or substituting it. The oxygen reduction reaction (ORR) suffers of several kinetic problems that were well described previously [1], [2], [3]. The situation gets even worse when working in neutral media in which H^+^ and OH^−^ that are main participants within the reaction are in an extremely low concentration (10^−7^ M) [6], [7]. Therefore, the reaction kinetic needs to be accelerated with supplement of catalysts. Biotic and abiotic catalysts are usually used in neutral conditions. Biotic catalysts are mainly enzymes and bacteria. Despite enzymes are extremely active and selective in neutral media [8], [9], [10], [11], they are expensive and not durable in harsh and polluted environments [12]. Bacteria catalysis for ORR is slow and electron transfer mechanisms are not fully understood [13], [14], [15], [16]. Abiotic catalysis instead is more used and based of utilization of high surface area carbonaceous materials, platinum group metal (PGM) catalysts and platinum group metals-free (PGM-free) catalysts. Few reviews summarize the achievements on the catalysis in neutral media [17], [18], [19], [20], [21], [22]. The first choice is quite utilized lately due to the material low cost, durability and the relatively high electrocatalytic activity towards ORR [21], [22], [23], [24], [25], [26], [27], [28]. In the recent years, activated carbon (AC) is by far the most adopted catalyst in microbial fuel cells (MFCs). Platinum catalyst was heavily adopted for MFCs application but recently abandoned due to the high cost that hinder large scale applications and then MFC commercialization and also due to the low durability in polluted environment containing anions such as sulfur, chloride, etc [29], [30], [31], [32]. The application of PGM-free catalysts for MFCs applications had a steep increase in the recent years due to the material low cost and higher ORR catalytic activity compared to AC and Pt [1], [17].

Different categories of PGM-free named i) metal oxides [33], [34], [35], [36], [37]; ii) Fe, Co, Ni metal center macrocyclic organic compounds [38], [39], [40], [41], [42], [43], [44], [45], [46]; iii) metal and organic compounds subject to high temperature treatment [47], [48], [49], [50], [51], [52], [53], [54], [55], [56] have been largely explored and investigated. In more details, the first category is composed by materials based on metal oxides with transition metals such as Fe, Co, Ni, Mn, etc [33], [34], [35], [36], [37]. The second category is based on macrocyclic organic compounds with Fe, Co, Ni, Mn, etc as metal center integrated in the structure [38], [39], [40], [41], [42], [43], [44], [45], [46]. The main organic structures are porphyrins and phthalocyanine and this type of PGM-free does not go through a high temperature (pyrolysis) process. The last category is a synthetic method that adopt high-temperature in which the precursors (organic based and metal based) are pyrolyzed at a temperature above 800–900 °C [47], [48], [49], [50], [51], [52], [53], [54], [55], [56].

Metal-nitrogen-carbon (M-N-C) PGM-free catalysts are based on atomically dispersed transition metal onto a nitrogen rich carbon substrate. Several successful examples with excellent performance are presented in literature utilizing Fe [38], [51], [57], [58], [59], [60], Co [61], [62], Ni [33], [34], [35], [36], [37], [38], [39], [40], [41], [42], [43], [44], [45], [46], [47], [48], [49], [50], [51], [52], [53], [54], [55], [56], [57], [58], [59], [60], [61], [62], [63] and Mn [34], [35]. The catalysts are then incorporated into air-breathing cathodes and integrated into MFCs. The high durability of PGM-free catalysts working in MFCs was also presented [29], [30], [51], [64]. It was recently shown that PGM-free catalyst can be mixed with AC and polytetrafluorethylene (PTFE) and pressed onto a current collector. PTFE is used as binder and is preferred to Nafion due to the much lower cost and its hydrophobic properties that benefit the performance. Lately, this solution is actually the most used due to the positive combination of cathode structure and high catalyst activity [29], [30], [64], [65]. In fact, AC/PTFE pellet-type air breathing configuration enhances the three phase interface (TPI). Moreover, superior electrocatalytic activity of PGM-free catalysts was also shown using rotating ring disk electrode (RRDE) [64], [66].

To the best of our knowledge, till now, there is no work presented in literature in which different PGM-free catalyst loadings added to the AC/CB/PTFE matrix are presented. In this investigation, the electrocatalytic activity of air-breathing cathode having different Fe-based catalyst loadings that varied between 0.25 mgcm^−2^ till 10 mg cm^−2^ were studied. Electrochemical performance as well as cost analysis are here presented in order to optimize the cathode performance for utilization in MFCs.

## 2 Materials and method

### 2.1 Catalyst preparation

Fe-AAPyr cathode catalysts working in MFC was previously reported [54], [66], [67], [68], [69]. Surface chemistry and morphology of the catalyst were deeply investigated in previously reported literature [29], [54], [66], [67], [68], [69]. Briefly, the catalyst was prepared utilizing sacrificial support method (SSM). Particularly, iron nitrate (Fe(NO_3_)_3_*9H_2_O) and aminoantipyrine were mixed with a dispersion of silica (Cab-*O*-Sil™ LM150, ∼200 m^2^ g^−1^) used as a template and then manually grounded using a mortar and a pestle. The sample was then inserted into a furnace in which the temperature was increased with a ramp rate of 25 °C min^−1^. When reached the temperature of 950 °C, the temperature was kept constant for 30 min and pyrolysis took place. The entire heat treatment was done in inert atmosphere utilizing a constant flow rate (100 mLmin^-1^) of Ultra High Purity (UHP) nitrogen. The silica used as sacrificial support was then etched using a dilute solution of HF (20%wt). The catalyst was then washed several times using DI water till neutral pH was reached. The catalyst was then dried at 85 °C to remove the excess in water.

### 2.2 Rotating ring disk electrode experiments

Rotating Ring Disk Electrode (RRDE) experiments were carried out using a glassy carbon electrode (Pine Research, USA) with polycrystalline Pt outer ring. An ink was then prepared and applied on the disk electrode. The ink was formulated using 8.5 parts of IPA:H_2_O (isopropanol:water 1:4 ratio) mixture and 1.5 part of 0.5 wt% Nafion with 5 mg of only Fe-AAPyr catalyst. The obtained mixture was ultrasonicated and then shaken for 4 min and 3 min respectively (three times each procedure). A pipette was used for drop casting the ink onto the disk that was then naturally dried in atmospheric environment. Eight different loadings were used during the RRDE investigation. The electrolyte used was 0.1 M potassium phosphate buffer (K-PB) electrolyte solution (pH 7.5) that was inserted into an electrochemical cell and vigorously purged with oxygen for over 20 min. This type of buffer was used to keep the pH stable at 7.5 during the duration of the tests and keep a “clean” solution avoiding the presence of sulfur and other pollutants that might interact directly with the catalyst diminishing its performance in RRDE. Linear sweep voltammetries (LSVs) were run at a scan rate of 5 mVs^−1^ in the potential range of 500 mV/-700 mV (vs Ag/AgCl). The electrode was kept at constant rotation speed of 1600 RPM. Pine bichannel potentiostat was used with a graphite rod as counter electrode and Ag/AgCl electrode (3 M KCl) as the reference electrode.

The disk current (I_disk_) and the ring current (I_ring_) were obtained and used to evaluate the hydrogen peroxide produced (%H_2_O_2_) using the following equation (1) (eq. (1)):

(1) $$\%H_{2}O_{2}=\;\frac{200\;\times \;\frac{I_{ring}}{N}}{I_{disk}+\frac{I_{ring}}{N}}$$

The number of electron transferred (n) can be also calculated using equation (2) (eq. (2)):

(2) $$n=\frac{4I_{disk}}{I_{disk}+\frac{I_{ring}}{N}}$$

N is the collection efficiency that was 0.43 as reported by the supplier.

### 2.3 Cathode preparation

Fe-AAPyr catalyst was then incorporated within an air-breathing cathode. The preparation was described previously in details [54], [64]. Particularly, activated carbon (AC, SX Ultra Norit, Sigma Aldrich), carbon black (CB, acetylene 50% compressed, Alfa Aesar) and polytetrafluorethylene (PTFE, 60% emulsion, Sigma-Aldrich) with a percentage in weight of 70%:10%:20% respectively were mixed within a grinder and milled for few minutes. The obtained mixture was then mixed vigorously with the catalyst in different concentration. The total weight added on the circular pellet die was 500 mg. This was due to the fact that a constant total loading of 50 mgcm^−2^ was used in order to keep the thickness of the air-breathing cathode constant within the different mixtures. Eight different loading were investigated as reported in Table 1. Each mixture was then pressed on a stainless steel mesh used as current collector at 2 mT for 5 min as previously described [54], [64].

**Table 1:** Cathode composition in terms of catalyst loading.

Fe-AAPyr	AC/CB/PTFE	total weight	catalyst loading
mg	mg	mg	mgcm^−2^
1	499	500	0.1
2.5	497.5	500	0.25
5	495	500	0.5
10	490	500	1
20	480	500	2
40	460	500	4
80	420	500	8
100	400	500	10

### 2.4 Linear sweep voltammetry in “clean” environment

After the electrodes fabrication, the cathodes were screwed to a lateral hole of the glassy single chamber microbial fuel cell (MFC) exposing the active part to the solution and the stainless steel (SS) mesh to the air. The glassy MFC was used as electrochemical cell. The cathodes were left in contact with the electrolyte (0.1 M K-PB) over night and then cathode polarization curves were run. In fact, linear sweep voltammetries (LSVs) were run using Biologic SP-50 potentiostat from open circuit voltage (OCV) till −0.4 V (vs Ag/AgCl) at scan rate of 0.2 mVs^−1^. Three-electrode configuration was used with the cathode acting as working electrode, Ag/AgCl as reference electrode and a titanium wire (>2 m in length) as counter electrode. To avoid undesired losses, a homemade Luggin-Haber capillary was used to bring the reference as close as possible to the cathode electrode. Measurements were done in triplicate and data reported in function of the cathode area exposed to the electrolyte (2.8 cm^2^).

### 2.5 Microbial fuel cell analysis

In parallel, after the fabrication, the cathodes were mounted on lateral hole of the glassy single chamber microbial fuel cell (MFC) with empty volume of 125 mL and exposed to 0.1 M K-PB overnight. The following day, the solution was changed into 50% 0.1 M K-PB and 50% activated sludge (AS) from a local civil treatment plant (Southside Wastewater Reclamation Plant, Albuquerque, NM, USA). This choice was dictated by three main reasons: i) the mixture of 0.1 M K-PB and AS had an overall solution conductivity that was similar to the 0.05 M K-PB and therefore the data obtained in this study can be compared with existing literature; ii) the presence of 50% AS simulate real environmental conditions containing potential pollutants and bacteria that might interfere negatively with the catalyst; iii) the activated sludge contains elements and compounds that are necessary for the bacterial growth and survival and therefore beneficial for the anode electrode. Anodes from existing well-working MFCs were transferred to the new MFCs. MFCs were left in open circuit voltage for at least 3 h to allow the voltage stabilization. Electrochemical experiments were then performed utilizing two Biologic SP-50 potentiostats in which one potentiostat was used to measure the overall polarization curves (V-I) and the other potentiostat was utilized to measure the potential profiles of the two electrodes during the polarization curves. Data are reported considering the cathode area exposed to the solution that was 2.8 cm^2^.

## 3 Results and discussion

### 3.1 RRDE results

Oxygen reduction reaction (ORR) is the reaction occurring at the cathode when oxygen is used as the final electron acceptor. ORR can follow two pathways in function of the electrolyte pH. Considering acidic media, the pathways can be different following: i) a direct 4e^−^ transformation with the reduction of O_2_ to H_2_O; ii) a 2e-transformation with the reduction of O_2_ to H_2_O_2_; or iii) a 2x2e^−^ transformation with the reduction of O_2_ to H_2_O_2_ that is then chemically or electrochemically transformed to H_2_O. Similar electron transfer mechanisms (4e^−^, 2x2e^−^ and 2e^−^) can be found considering alkaline media. The pathways followed can be different: i) a direct 4e^−^ transformation with the reduction of O_2_ to OH^−^; ii) a 2e^−^ transformation with the reduction of O_2_ to HO_2_^−^ and OH^−^; or iii) a 2x2e^−^ transformation with the reduction of O_2_ to HO_2_^−^ and OH^−^ that is then chemically or electrochemically further transformed to OH^−^. A direct 4e^−^ transfer mechanism is preferred and it is more efficient compared to a 2x2e-mechanism. A 4e^−^ transfer mechanism is also more desirable compared to a 2e^−^ mechanism since double oxidant is needed to complete the red-ox reaction. In microbial fuel cells, the catalysts for the anode are biotic and based on electroactive bacteria and therefore peroxide is undesired since it can act as a disinfectant (bactericidal) and negatively affect the biofilm.

**Fig. 1:**
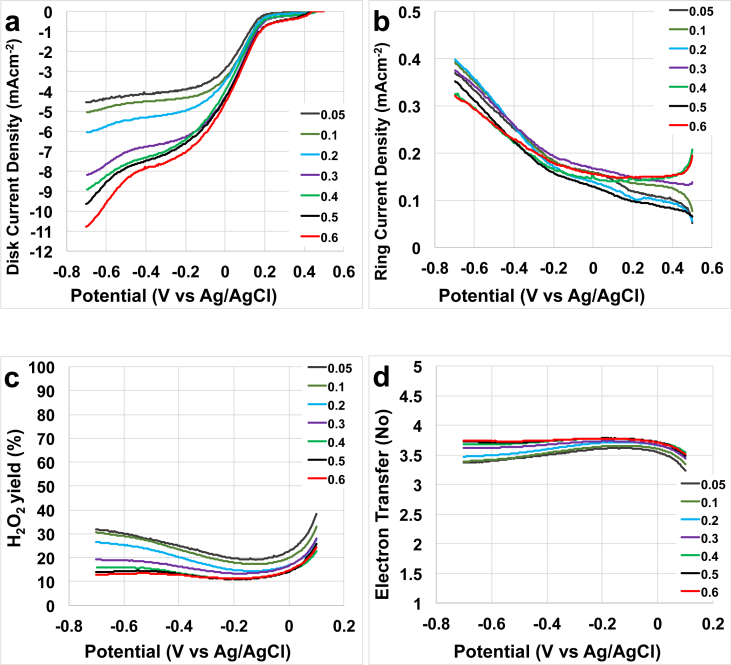
Disk current (a), ring current (b), peroxide yield (c) and electron transfer number (d) at rotating speed of 1600 rpm for catalyst loading varying from 0.05 to 0.6 mgcm^−2^.

The disk current recorded in the experiments showed that the increase in catalyst loading lead to an increase in the half-wave potential and the limiting current (Fig. 1a). The onset potential was independent from the catalyst loadings and was roughly 0.30–0.35 V (vs Ag/AgCl) (Fig. 1a). The steady-state value of the limiting current density achievable in aqueous electrolyte is a function of concentration of dissolved molecular oxygen and cannot exceed 7.5 mAcm^−2^ under standard conditions (1 Bar total pressure and room temperature). In this experiment, however, a substantially higher limiting current up to ≈11 mAcm^−2^ has been observed for the catalyst loadings above 0.3 mgcm^−2^ (Fig. 1a). This indicates that the catalyst reduces molecular oxygen by complex mechanism, which includes a hydrogen peroxide formation step, and heterogeneous (non-electrochemical) decomposition of the hydrogen peroxide inside the porous matrix of the catalyst gives rise of the local oxygen concentrations within immediate proximity of the oxygen reduction active sites, thus resulting in current densities values higher that the ones expected if only the molecular oxygen from the solution would have been electro-reduced.

The ring current was also measured and it increased with the decrease in potential (Fig. 1b). The peroxide is an intermediate of the reaction therefore it is undesired and it makes the reaction less efficient compared to a direct 4e-transfer mechanism. The peroxide yield was measured and it decreased with the catalyst loading (Fig. 1c). This indicates that higher loading creates a thicker catalyst layer and therefore the peroxide produced is consumed within the thicker layer. In fact, at −0.7 V (vs Ag/AgCl), loadings of 0.05–0.1 mgcm^−2^ produced roughly 30% of peroxide, instead higher loadings (0.5–0.6 mgcm^−2^) produced 12–14% of peroxide (Fig. 1c). Consequently, also the electron transfer mechanism was related with the quantity of catalyst applied on the disk (Fig. 1d). In fact, at −0.7 V (vs Ag/AgCl), the number of electron transferred at loadings of 0.05 and 0.1 mgcm^−2^ was ≈3.4. This number increased up to ≈3.75 at higher loadings (0.5–0.6 mgcm^−2^) (Fig. 1d). A low but not negligible peroxide production and a general constant increase in performance with the catalyst loading might indicate a 2x2e-transfer mechanism rather than a preferred direct 4e^−^.

### 3.2 Cathode polarization curves run in “clean” electrolyte media

The RRDE technique is used worldwide to determine the catalysts kinetics. RRDE tests are done in operating conditions in which the oxygen supply comes from the dissolved oxygen into the liquid electrolyte. The LSVs obtained through the RRDE experiments can indicate which catalyst had higher performance evaluating the electrochemical parameters of interest such as half-wave potential and limiting current. Different conditions are instead established when the catalyst is integrated into the air breathing cathode design. This type of configuration is specifically designated to enhance a three-phase interface (TPI) in which the oxygen is supplied both through gas phase and through liquid phase (dissolved oxygen). Particularly, the gas phase is delivered to the catalytic sites through the hydrophobic pores inside the cathode structure instead the dissolved oxygen permeates through the hydrophilic pores of the cathode. As the two techniques consider different operating conditions, a direct comparison cannot be done. Theoretically, as the oxygen is supplied in gas phase, the performance of the air-breathing cathode should be higher compared to the results on the RRDE. Another difference that should be underlined is that only the catalyst (with the binder) is applied on the disk during RRDE measurements while in the air-breathing cathode, the catalyst has a loading of 0.1–10 mgcm^−2^ and it is incorporated into a matrix of AC/CB/PTFE with a loading of 40–49.9 mgcm^−2^. In the air-breathing cathode, the catalyst weight corresponds to a percentage between 0.2% and 20% of the total weight of the “black powder” mixture.

**Fig. 2:**
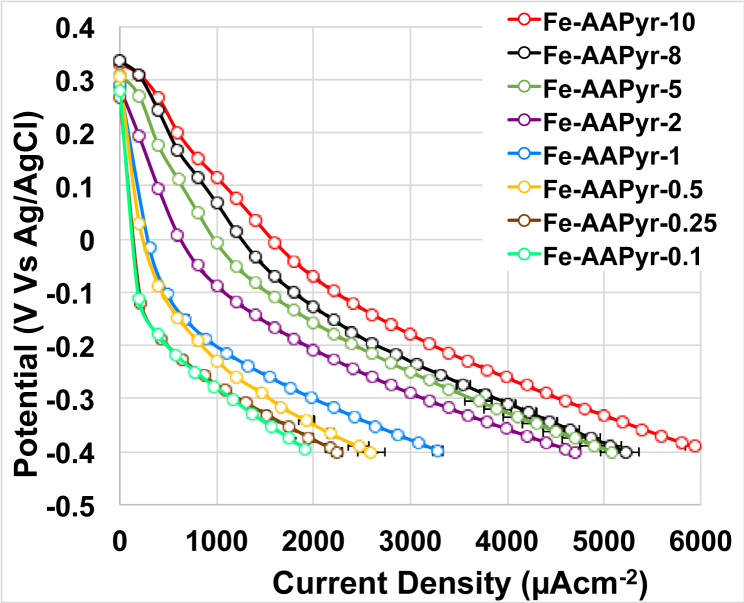
Polarization curves of the cathodes having different catalyst loadings in “clean” electrolyte.

LSVs were run for the air-breathing cathode in circumneutral pH (pH 7.5) after leaving the cathode in contact with the buffer solution overnight (Fig. 2). As expected, the open circuit potential (OCP) was similar independently from the catalyst loadings and measured 302 ± 22 mV (vs Ag/AgCl). As the theoretical ORR value at pH 7.5 is ≈ 576 mV (vs Ag/AgCl 3 M KCl), this particular cathode showed an initial activation overpotential between 252 mV and 294 mV (Fig. 2). The polarization showed that the higher electrocatalytic activity among the cathodes investigated was the one of the cathodes having higher catalyst loading (10 mgcm^−2^) (Fig. 2). At the contrary, the lower performance were recorded for the cathodes with lower catalyst loading (0.1 mgcm^−2^) (Fig. 2). The trend of enhanced performance with the increase of the catalyst loading within the cathode shown with the RRDE data follows the same trend identified in the air-breathing cathode tested in “clean” electrolyte.

### 3.3 Microbial fuel cell output

**Fig. 3:**
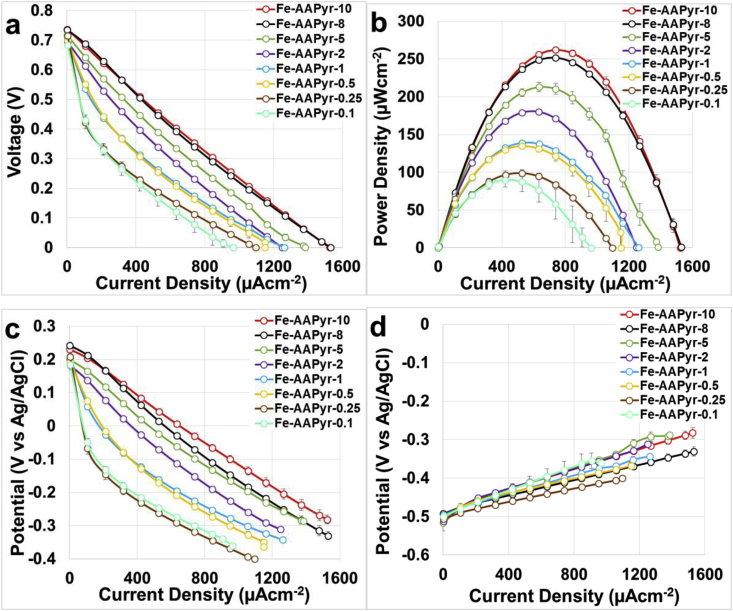
Overall polarization curves (a), power curves (b), cathode polarization (c) and anode polarization curves (d) of the MFCs having different cathode catalyst loadings.

Overall polarization curves (Fig. 3a), power curves (Fig. 3b) and single electrode polarization curves (Fig. 3c and d) are here presented. The polarization curves of the MFCs having cathodes with Fe-AAPyr had very similar OCV (Fig. 3a). The electrocatalytic activity increased with the increasing of catalyst loading and this can be detected by the enhancing of the slope of the polarization curve (Fig. 3a). Interestingly, the slopes of the polarization curves still remain approximately linear indicating that ohmic resistance is mainly present within the system. It was shown before that an increase in ionic strength of the electrolyte brings to higher performance without changing cathode composition [48], [64]. Power curves showed an increase in the peak production with the increase in the cathode catalyst loadings (Fig. 3b). Particularly, the power density obtained were 90 ± 9 μWcm^−2^ (252 ± 25 μW), 99 ± 5 μWcm^−2^ (277 ± 14 μW), 135 ± 1 μWcm^−2^ (378 ± 3 μW), 139 ± 3 μWcm^−2^ (389 ± 8 μW), 180 ± 1 μWcm^−2^ (504 ± 3 μW), 213 ± 6 μWcm^−2^ (596 ± 17 μW), 252 ± 2 μWcm^−2^ (706 ± 6 μW), 262 ± 4 μWcm^−2^ (734 ± 11 μW) for catalyst loading of 0.1, 0.25, 0.5, 1, 2, 5, 8 and 10 mgcm^−2^ respectively.

Therefore, we can conclude that the increase in catalyst loading at the cathode influences positively the MFC output. Single electrodes polarization curves showed that cathode performance were substantially enhanced by the increase in catalyst loading (Fig. 3c). Particularly, the increment in catalyst loadings enhanced the performance in the first 400 μAcm^−2^ of the cathode polarization curves diminishing the activation losses. Single electrodes polarization curves showed that anode polarization curves were similar but some differences can be detected (Fig. 3c). As the same anode electrodes were employed, the differences observed may be due to the variation in the ambient temperature since the tests were conducted in a laboratory with centralized temperature management, but no thermostatic control. It was noticed that the temperature during the entire experimental period varied within 5 °C (22.5 ± 2.5 °C), which is sufficient to affect the levels of performance; this is also in agreement with existing literature [70], [71]. Once again, both anode and cathode polarization curves did not suffer of any diffusion limitation therefore ohmic losses play an important role.

### 3.4 Cost analysis

**Fig. 4:**
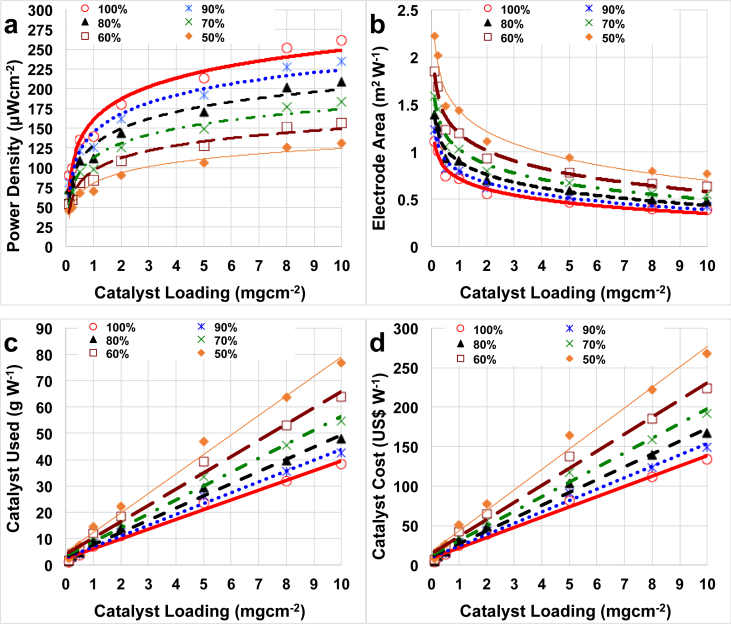
Peak of power density (a), electrode area need to generate a power of 1 W (b), catalyst used to generate a power of 1 W (c) and catalyst cost to generate 1 W (d) in function of the catalyst loading and the durability of the system.

Due to the low current/power generation produced by microbial fuel cells, enhancement in the electrochemical output is strongly desired as well as the containment of the costs that have to be minimized to make the system competitive compared to other low-power devices. The factors included in this cost analysis were: i) power produced (μWcm^−2^) vs. catalyst loading (mgcm^−2^); ii) surface area necessary to produce 1 W (m^2^ W^−1^) vs. catalyst loading (mgcm^−2^); iii) quantity of the catalyst used to produce 1 W (m^2^ W^−1^) vs. catalyst loading (mgcm^−2^); iv) cost of the catalyst used to produce 1 W (m^2^ W^−1^) vs. catalyst loading (mgcm^−2^). In this work, the maximum power peak was used for the analysis (100%) as well as it was assumed that performance might decrease over time due to the cathode/catalyst deterioration. Therefore, a reduction of 10%, 20%, 30%, 40% and 50% were also considered and in Fig. 4 those losses were expressed as the MFC was operating at 90%, 80%, 70%, 60% and 50% compared to the initial power peak (100%). It was assumed that the general deterioration was independent from the catalyst loading integrated in the cathode.

As mentioned before, the increase in catalyst loading at the cathode lead to an enhancement in the power obtained (Fig. 4a). The power peaks follows a logarithmic trend probably reaching a plateau after further increase in loading. In our experimental investigation, additional increase in loading was not possible because the cathode started to lose consistency and the adhesion to the stainless steel mesh was not proper. The initial peak of power densities (100%) changed between 90 μWcm^−2^ (catalyst loading of 0.1 mgcm^−2^) and 262 μWcm^−2^ (catalyst loading of 10 mgcm^−2^) (Fig. 4a). If an important drop of the performance quantified in 50% occurred, the performance would drop to 45 μWcm^−2^ (0.1 mgcm^−2^) and 131 μWcm^−2^ (10 mgcm^−2^) respectively.

For practical applications in which the overall power is the parameter considered, high power density can be explained into lower electrode area necessary to achieve the same absolute power and make the system more compact (Fig. 4b). In this case, the overall power of 1 W was chosen as general power output for simplicity in the calculation. As it can be noticed in Fig. 4 b, considering the initial peak of power density (100%) for each loading, 1.11 m^2^ of electrode area was necessary to achieve 1 W of overall power with loading of 0.1 mgcm^−2^. This value was 2.9 higher (0.38 m^2^) compared to the electrode needed if the selected loading was 10 mgcm^−2^. As the deterioration of the cathode/catalyst might occur a larger cathode electrode surface area should be deployed. Considering the catalyst loading of 0.1 mgcm^−2^, cathode geometric area of 1.23 m^2^, 1.39 m^2^, 1.59 m^2^, 1.85 m^2^ and 2.22 m^2^ would be necessary if the power produced was 90%, 80%, 70%, 60% and 50% respectively compared to the initial power produced. In parallel, with the higher catalyst loading investigated, the cathode geometric area necessary would be 0.42 m^2^, 0.48 m^2^, 0.55 m^2^, 0.64 m^2^ and 0.76 m^2^ respectively, still much lower compared to low catalyst loading. Therefore, if the main parameter to consider is the space, in order to achieve the highest power output, higher catalyst loading on the electrode should be preferred.

Despite PGM-free estimated cost is relatively low, the cost is much higher compared to commercially available AC that is roughly 0.1 US$g^−1^ (Sigma Aldrich). In a previous work, Fe-based cost considering just the raw material purchased from Sigma Aldrich and avoiding the capital cost (e.g. electricity, human labor, capital cost, etc) was roughly estimated to be 3.5 US$g^−1^ [72]. Both catalysts used (Fig. 4c) and catalyst cost (Fig. 4d) in order to generate 1 W of power increased with the catalyst loading utilized. Particularly, if the catalyst loading was 0.1 mgcm^−2^, 1.11 g is necessary to reach 1 W considering the initial power peak (Fig. 4c). This amount increases to 1.23 g, 1.39 g, 1.59 g, 1.85 g and 2.22 g if the performance decrease over time by 10%, 20%, 30%, 40% and 50% respectively (Fig. 4 c. Much higher catalyst quantity (38.3 g) is necessary to achieve 1 W (100%) if the catalyst loading considered is 10 mgcm^−2^ (Fig. 4c). This quantity would increase to 42.4 g, 47.7 g, 54.5 g, 63.6 g and 76.3 g with the MFC performing 90%, 80%, 70%, 60% and 50% respectively the initial value (Fig. 4c).

The catalyst cost increased significantly following a linear trend from 3.9 US$W^−1^ (loading 0.1 mgcm^−2^) to 38.9 US$W^−1^ (loading 2 mgcm^−2^) till 134.0 US$W^−1^ (loading 10 mgcm^−2^). If a reduction of performance of 10% occurred, the cost would increase to 4.3 US$W^−1^ (loading 0.1 mgcm^−2^) to 43.2 US$W^−1^ (loading 2 mgcm^−2^) till 148.4 US$W^−1^ (loading 10 mgcm^−2^). If the reduction was more dramatic and quantified in 50%, the cost would increase to 7.8 US$W^−1^ (loading 0.1 mgcm^−2^) to 77.8 US$W^−1^ (loading 2 mgcm^−2^) till 268 US$W^−1^ (loading 10 mgcm^−2^). It can be noticed that switching from double chamber to single chamber MFC, the electrochemical performance increased significantly and therefore the overall cost due to the catalyst decreased importantly.

If current/power generation is the only objective of the microbial fuel cell and reducing the cost is not a priority, higher performance are achieved utilizing high catalyst loading. A more serious interpretation should be done if also the costs are important to be maintained low. At the moment, the catalyst addition certainly helps the performance improvements but the cost is still high. Therefore, further studies should consider decreasing at least one order of magnitude the catalyst cost fabrication.

## 4 Conclusions

Electrocatalytic activity of Fe-AAPyr was investigated in RRDE and the results showed an increase in the limiting current and half wave potential with the increase in catalyst loading. The increase in loading lead also to a decrease in peroxide formation and an increase in the number of electrons transferred. A 2x2e^−^ mechanism is suggested due to the production of peroxide detected. Once the catalyst was integrated inside the air-breathing cathode and tested in operating MFCs, the performance increased with the catalyst loading. Power density varied between 90 ± 9 μWcm^−2^ and 262 ± 4 μWcm^−2^ with the lowest (0.1 mgcm^−2^) and the highest (10 mgcm^−2^) catalyst loading investigated. Polarization curves showed that the performance variations were mainly due to the cathode. If the main goal of the microbial fuel cell is to produce high power/current, high catalyst loading is suggested. At the contrary, the catalyst cost is still high and different preparation methods have to be explored to decrease the catalyst cost.
